# Strategic Priorities for Advancing Eating Disorder Risk Reduction: A Narrative Review

**DOI:** 10.1002/eat.70124

**Published:** 2026-05-05

**Authors:** Hannah K. Jarman

**Affiliations:** ^1^ School of Psychology, Faculty of Health Deakin University Geelong Victoria Australia; ^2^ SEED Lifespan Strategic Research Centre, School of Psychology, Faculty of Health Deakin University Geelong Victoria Australia

**Keywords:** eating disorders, prevention, risk reduction

## Abstract

Despite decades of advances in treatment, eating disorders continue to impose substantial individual and societal burden, underscoring the need for earlier and more effective risk reduction. Prevention research has expanded considerably, producing a wide range of approaches that target modifiable risk factors, build individual coping skills, intervene within schools and families, leverage digital delivery formats, and, increasingly, consider policy and structural levers. Although many programs demonstrate small‐to‐moderate improvements in established risk factors, population‐level impact has been limited. Effects are often modest, implementation is inconsistent, and scalability remains constrained. This narrative review synthesizes contemporary eating disorder risk reduction efforts across four interconnected dimensions that shape impact: mechanisms targeted, level of focus (from individual to population), personalization and cultural adaptation, and delivery and scalability. Across these domains, critical gaps emerge, including narrow mechanistic focus, imprecise risk stratification, underrepresentation of diverse populations, and limited integration of implementation science and economic and policy evaluation. This review argues that advancing eating disorder risk reduction requires a shift from efficacy‐focused intervention development toward coordinated, multilevel, and implementation‐informed strategies that prioritize precision, equity, system integration, and policy engagement. Such reorientation is essential if eating disorder risk reduction is to achieve meaningful and sustained population‐level impact. Drawing on these findings, this review outlines a set of strategic priorities for advancing eating disorder risk reduction at scale.

## 1 Introduction

Eating disorders are serious and costly to individuals and society. Although progress has been made in treatment, many interventions are delivered only after symptoms have become entrenched, when recovery is more difficult and resource‐intensive. Even with advances in treatment, incidence has remained stable or increased in some regions (Hay et al. 2023). As such, reducing risk earlier in the trajectory appears critical to reduce prevalence, burden, and healthcare costs.

Prevention research has grown considerably over the past two decades. Existing programs vary widely in their targets, delivery modes, and reach, and are typically classified as universal (i.e., aimed at a whole population; e.g., Happy Being Me; Richardson and Paxton 2010; Media Smart; Wilksch and Wade 2009), selective (i.e., high‐risk groups such as adolescents or individuals with body image concerns; e.g., Student Bodies; Jacobi et al. 2012; Body Project; Stice and Presnell 2007), or indicated (i.e., individuals with early symptoms such as binge eating, dietary restraint; e.g., Break Binge Eating; Linardon et al. 2022). Numerous reviews have evaluated the efficacy of prevention programs (e.g., Koreshe et al. 2023; Le et al. 2017; Watson et al. 2016), demonstrating small‐to‐moderate reductions in eating disorder risk factors and symptoms, particularly among adolescents and young adults and over shorter follow‐up periods. Despite this growing evidence base, effects are often modest and difficult to sustain at scale, with many programs demonstrating efficacy under controlled conditions yet struggling to translate into population‐level change.

Taken together, findings suggest that the challenge is not simply to develop more programs, but to rethink how prevention is conceptualized, assessed, and implemented. Yet few reviews have synthesized prevention across levels—from individual mechanisms to structural and policy‐based approaches—or interrogated reach, sustainability, and system integration. Without such integration, prevention risks remaining fragmented and limited in impact.

This narrative review seeks to offer a brief, focused, and integrative synthesis of current eating disorder risk reduction approaches designed to reduce the likelihood of eating disorder symptom emergence or diagnosis. In this context, eating disorder risk reduction refers to prevention delivered prior to disorder onset (i.e., primary prevention). Rather than providing an exhaustive systematic review, this review draws on key evidence, prioritizing meta‐analyses, systematic reviews, and key empirical studies in the field, including randomized controlled trials and emerging approaches. Consistent with a narrative review approach, no formal systematic search, screening, or meta‐analytic procedures were undertaken. Instead, studies were selected based on their contribution to the field and relevance across four key dimensions shaping real‐world impacts: mechanisms targeted, level of focus (i.e., individual to population), personalization and adaptation, and delivery and scalability. This review examines what we have learned, identifies critical gaps, and highlights strategic priority areas, of which the latter is summarized in Table 1 to provide a clear agenda for advancing prevention at scale.

**TABLE 1:** Strategic priorities for advancing eating disorder risk reduction.

Domain	Current limitations	Strategic priorities	Example research or practice approaches
Risk prediction and mechanisms	Narrow focus on isolated risk factors; limited precision in identifying risk	Develop multivariable, transdiagnostic risk models to enable precision prevention	Integrate transdiagnostic and eating disorder specific predictors Use AI/machine learning Collect large datasets by embedding measures in routine school/healthcare data
Active intervention ingredients	Multicomponent programs without clarity on which element(s) drive effects	Identify core intervention mechanisms	Conduct dismantling and factorial trials Test streamlined versions of programs for scalability
Level of focus (Individual → Policy)	Predominant focus on individual‐level change	Expand system‐ and policy‐level approaches	Whole‐of‐school programs Advocate for policy change Evaluate regulatory and public health policies
Cultural adaptation	Over‐reliance on White, Western, educated samples	Co‐design culturally safe and contextually relevant programs	Partner with marginalized communities to develop and evaluate interventions Develop and validate population‐specific measures
Personalization	One‐size‐fits‐all models; vague definitions of personalization/tailoring	Operationalize and test precision prevention models	Risk‐based stratification and adaptive/stepped‐care designs Digitally automated tailoring
Delivery and engagement	Challenges with engagement and sustainability	Optimize scalable, low‐burden formats	Micro‐interventions and single‐session formats Hybrid digital and human models Engagement optimization trials
Implementation and scalability	Heavy reliance on controlled efficacy trials	Embed implementation science within prevention	Hybrid effectiveness‐implementation designs Conduct pragmatic trials in real‐world settings
Economic evaluation	Limited integration of cost‐effectiveness data	Embed economic modeling into prevention research	Compare different prevention approaches or levels (e.g., individual vs. policy) Cost‐utility analyses

### 1.1 Understanding Risk and Etiology

Eating disorder risk reflects a complex interplay of biological vulnerability with psychological, social, and developmental influences (Culbert et al. 2015). Modifiable mechanisms, such as thin‐ideal internalization, body dissatisfaction, and dietary restraint, often emerge early and are shaped by peer, family, and media environments. However, the multi‐determined and heterogeneous nature of eating disorders means risk pathways and intervention approaches vary considerably.

An important consideration in evaluating effectiveness is how success is operationalized. Traditionally, reviews and meta‐analyses have determined success as statistically significant improvements in risk factors (e.g., Koreshe et al. 2023). A small number of trials demonstrate reduced onset in higher‐risk groups (Stice et al. 2021), yet practical implications for real‐world impact are less examined. Instead, this review argues that success should also be interpreted in relation to reach, cost, and scalability.

### 1.2 Dimension #1: Mechanisms Targeted

Prevention programs draw on diverse theoretical frameworks, targeting distinct risk factors. Dissonance‐based interventions challenge thin ideals; cognitive behavioral therapy (CBT) and third‐wave approaches target maladaptive cognitions and emotion regulation; media literacy builds critical appraisal skills. Effects vary by population: systematic reviews and meta‐analyses have shown that media literacy tends to produce small effects in universal samples, whereas more intensive approaches show stronger outcomes in selective groups (Le et al. 2017; Watson et al. 2016). Larger effects, however, often require greater resources, creating a trade‐off between efficacy and scalability.

Many programs target a narrow set of proximal risk factors (e.g., body dissatisfaction, thin‐ideal internalization; Koreshe et al. 2023) despite evidence that risk arises from multilevel, interacting influences (e.g., Culbert et al. 2015). For instance, Stice et al. (2011) identified multiple, qualitatively distinct risk pathways to eating disorder onset, noting that prevention efforts should be tailored to distinct risk profiles rather than a single risk factor. This complexity of risk limits our ability to accurately predict who is most likely to develop symptoms, meaning selective and indicated approaches may fail to reach those at highest risk or may allocate limited resources inefficiently. Improving prediction is therefore central to enhancing the precision, cost‐effectiveness, and impact of prevention efforts.

Evidence has shown that risk prediction performs best when considering both transdiagnostic and eating disorder‐specific predictors (Mitchison et al. 2023), highlighting the need to utilize multivariable approaches. Artificial intelligence (AI) approaches, including machine learning, may offer an opportunity to strengthen predictive accuracy (McClure et al. 2025). However, a key challenge here is collecting the required large datasets, which are often time‐intensive and costly. Embedding validated measures within routinely collected healthcare or school data (Obeid et al. 2025) may offer promise. Alternatively, thoughtful studies that utilize cross‐institutional and international collaboration within eating disorders may also provide a feasible path forward by pooling data and expertise to support large‐scale data collection and modeling efforts.

Eating disorder risk factors have been found to operate in combination rather than distinctly, suggesting that multicomponent interventions may be advantageous (Jarman et al. 2024). However, evidence directly comparing multicomponent and single‐component approaches is limited, with meta‐analyses constrained by too few multicomponent studies to permit robust subgroup comparisons (Le et al. 2022). Consequently, although some emerging programs integrate multiple components, it remains unclear which elements are most (or least) effective and for whom. Dismantling studies are therefore an important next step to determine active intervention ingredients, allowing for more streamlined interventions or various intervention versions. Furthermore, these approaches would provide insights into the underlying processes which influence the trajectory, thereby better informing current models.

### 1.3 Dimension #2: Level of Focus

Prevention has traditionally focused on addressing the individual through skills‐based programs such as mindfulness, self‐compassion, and lifestyle modification. However, in recognition of the role the environment plays in eating disorder risk, some programs also seek to address system‐level factors (Burton et al. 2025). For example, schools, families, and health professionals shape children's body image and hence have been a target of focus for eating disorder risk reduction. Intervening within these ecosystems can shift the norms, attitudes, and behaviors that underpin risk and enable earlier identification and support.

Whole‐of‐school models illustrate this approach. For example, Butterfly Body Bright is a whole‐of‐school program that aims to support schools and families to reduce risk factors and enhance protective factors in primary school aged children. The program contains staff training, curriculum‐aligned lessons from Prep‐Year 6, school culture guidelines, and family resources. Butterfly Body Bright has shown preliminary evidence of significant improvements after just one lesson on body image, body appreciation, confidence to deal with appearance‐based teasing, and help seeking if needed (Damiano et al. 2024); however, this trial did not include a control group and thus cannot yet be compared to evidence of existing school‐based programs.

Embedding such programs into existing systems, like schools, will likely enhance sustainability over time. Similarly, parent interventions for body image have also been translated into community child health services (e.g., Norton et al. 2022). To ensure implementation efforts are successful, collaboration and partnership across cross‐sector researchers, eating disorder organizations, individuals and families with lived experience, etc. is essential (Ciao et al. 2024).

Public health initiatives and policies are also an integral lever to reducing eating disorder risk given their significant potential for reach and for reducing inequities. Although regulatory and educational policies have been proposed, only a small number are empirically evaluated. For example, among 74 policy recommendations to prevent disordered weight control behaviors identified by Raffoul et al. (2023), only one empirical study was found that evaluated the implementation of such a policy, highlighting a critical knowledge gap.

Policy reform is often protracted and uncertain but offers greater population‐level impact than individual interventions. For those who wish to advocate for such policies, the triggers‐to‐action framework helps guide and prioritize policy translation (Austin 2016). It underpins important work conducted at the world‐leading Strategic Training Initiative for the Prevention of Eating Disorders (STRIPED; www.hsph.harvard.edu/striped), whose research and advocacy have helped drive critical policies in the United States, including New York's ban on over‐the‐counter diet pills and muscle‐building supplements for minors.

One useful approach when advocating for policy change is to determine the cost‐effectiveness of different approaches, given its influence on decision‐making among policymakers and other interested groups (Whiteford and Weissman 2017). For example, a review by Long et al. (2022) examined the cost‐effectiveness of various public health approaches to prevention (e.g., school/primary care‐based screening, excise tax on over‐the‐counter diet pills). Although all approaches showed cost‐effectiveness compared to normal practice, the largest savings were found for school‐based universal prevention ($1102 per person). Despite the promise of these savings, the capacity to embed and sustain such programs within existing systems remains a critical implementation challenge that must be addressed to realize their economic potential.

### 1.4 Dimension #3: Personalization and Adaptation

A large focus of eating disorder risk reduction has been among high‐risk samples, particularly student and female populations. The Body Project, a peer‐led cognitive dissonance program, is among the most extensively researched in these populations. Following established efficacy at reducing eating disorder risk factors and onset (Becker and Stice 2017), the Body Project has since been adapted across a diverse range of at‐risk populations. For example, in athletes, where risk has long been characterized in relation to the female athlete triad (i.e., low energy availability, menstrual dysfunction, and osteoporosis), the Body Project has been adapted into the Female Athlete Body Project, the Male Athlete Body Project, and the Young Athlete Body Project. These adaptations have shown preliminary efficacy and acceptability in adults and adolescents (e.g., see Burton et al. 2025). Among cisgender gay and bisexual men, a randomized controlled trial of the PRIDE Body Project demonstrated feasibility, acceptability, and efficacy for reducing risk up to 1‐year later (e.g., Almeida et al. 2024). There is also an increasing number of prevention programs which target individuals in a larger body and/or with diabetes, with the most consistent improvements in risk and symptoms observed in the Diabetes Body Project (see D'Silva et al. 2026 for a systematic review). While these adaptations represent important progress, they are largely derived from a single program and more needs to be done to develop or adapt other programs to meet the needs of other diverse samples, cultures, and contexts.

More broadly, prevention programs have typically included White, Western, and relatively advantaged samples, limiting generalizability of findings and increasing health inequalities. Indeed, different risk factors may operate in more diverse populations and current approaches may not be suited to their individual needs. Given the distinct needs and structural barriers faced by at‐risk groups, meaningful adaptation requires co‐design approaches—that is, actively partnering with community members and stakeholders throughout program development. Co‐design is crucial because it ensures interventions are culturally safe, contextually relevant, and acceptable to the populations they aim to serve, thereby enhancing engagement, feasibility, and real‐world impact (Miskovic‐Wheatley et al. 2025). Relatedly, appropriate measures are needed to determine effectiveness among distinct groups, which may require population‐specific tools (Alexander et al. 2024).

Eating disorders are heterogeneous in their presentation, risk pathways, and response to intervention, meaning a one‐size‐fits‐all approach is not sufficient. More personalized approaches (e.g., risk profiling or targeted screening) are needed, yet despite longstanding calls, there has been a dearth of action, with practice lagging behind theory. As with broader issues around how we define prevention success, progress may be limited by how we define personalization, ranging from engagement features (e.g., notifications) to content tailoring or risk‐based targeting. Authors should specify clearly how the program has been personalized. However, real‐world constraints like time, staffing, and resources often limit the feasibility and scalability of personalized approaches.

These challenges may be overcome through various approaches. For example, personalization may be automated through digital interventions (e.g., via screening and engagement data). Nacke et al. (2024) demonstrated the feasibility of delivering a suite of online screening and tailored prevention/intervention programs to reach different risk groups. Combining universal programs with targeted additional modules for higher‐risk groups or adaptive intervention designs such as stepped‐care or just‐in‐time support also offer promise, highlighting the potential for digitally enabled precision prevention (Linardon and Torous 2025).

### 1.5 Dimension #4: Delivery and Scalability

Physical, geographical, and financial barriers have been shown to limit in‐person access to interventions. To enhance reach and scalability, researchers have turned their attention to online delivery, with programs provided via e‐health, websites, apps, or chatbots offering more equitable support. Online programs have demonstrated efficacy for reducing eating disorder risk factors in selective and indicated samples (e.g., Linardon et al. 2025). While these delivery formats offer convenience, they also face challenges with sustained engagement over time (Liu et al. 2026). As such, researchers have started to explore single‐session and micro‐interventions (e.g., Schleider et al. 2023; Thompson et al. 2026). A recent meta‐analysis and systematic review suggests that single session interventions show promise in reducing eating disorder risk factors (Negi et al. 2026).

Chatbots are also emerging as an alternative modality to deliver personalized prevention at scale, including both rule‐based chatbots (i.e., pre‐scripted automated responses) and generative AI chatbots (Gen‐AI; i.e., systems that generate responses based on training datasets). For example, Heinz et al. (2025) conducted a randomized controlled trial of a 4‐week Gen‐AI chatbot, Therabot, for mental health (including adults at high risk for feeding and eating disorders). Participants reported significantly greater reductions in eating disorder risk at 4‐ and 8‐week follow‐up relative to control. However, despite the potential of these emerging spaces, caution is warranted given concerns regarding safety, accuracy, privacy (Craddock et al. 2026), particularly for generative AI systems.

Given that typical eating disorder onset occurs between 15 and 23 years (Solmi et al. 2024), many programs target children, adolescents, and young adults. Such programs often deliver universal content in school and university settings, reaching larger numbers at a relatively low cost. Systematic reviews and meta‐analyses have shown small‐to‐moderate effects of dissonance‐based and media literacy interventions on eating disorder risk factors and symptoms in university settings (Harrer et al. 2020) and schools (Berry et al. 2025). These may be delivered in‐person by the classroom teacher, a peer‐leader, or a researcher/clinician, or may also be offered online, each with differing levels of necessary resourcing and potential for scalability. Further research is needed to explore which approaches offer the most cost‐effective and sustainable outcomes.

Another key barrier to the scalability of eating disorder risk reduction is reliance on efficacy trials in highly controlled settings, which often fail to reflect real‐world contexts. This creates a research–practice gap, delaying translation and limiting population‐level impact. Integrating implementation science within prevention research, particularly hybrid effectiveness–implementation designs (Curran et al. 2012), can address this by evaluating outcomes and implementation simultaneously. This allows researchers to assess not only *whether* a program works, but *how*, *for whom*, and in what contexts programs work sustainably. Embedding factors such as feasibility, acceptability, fidelity, cost, and system fit from the outset increases scalability and real‐world impact. For eating disorder risk reduction, this shift from efficacy‐first to implementation‐informed research is critical for achieving meaningful population‐level change.

## 2 Conclusion

Eating disorder risk reduction has generated a substantial and diverse evidence base, demonstrating that prevention can improve established risk factors and, in some cases, reduce onset. However, modest effect sizes, limited scalability, and persistent research–practice gaps suggest that progress at the population level has been constrained. Advancing the field will require moving beyond isolated efficacy trials toward more integrated and strategic approaches, including refining multivariable risk prediction to enable precision prevention, identifying active intervention ingredients, embedding programs within schools, healthcare systems, and communities, advocating for and evaluating policy levers, meaningfully co‐designing interventions with diverse populations, and incorporating implementation science from the outset to ensure feasibility, sustainability, and equity. Ultimately, the central challenge is not whether prevention works, but how to design, deliver, and scale it in ways that achieve meaningful and lasting population‐level impact.

## Author Contributions

**Hannah K. Jarman:** conceptualization, investigation, project administration, writing – original draft, methodology, visualization, writing – review and editing.

## Funding

The author has nothing to report.

## Lived Experience Involvement Statement

No specific efforts were undertaken to involve persons with lived experience in the study design or execution, or in the preparation of this manuscript.

## Disclosure

The author used ChatGPT‐5 to assist with proofreading of the final version of this manuscript. I confirm that all AI‐assisted content was carefully reviewed and edited to ensure accuracy and appropriateness.

## Conflicts of Interest

The author declares no conflicts of interest.

## Data Availability

Data sharing not applicable to this article as no datasets were generated or analysed during the current study.
